# Quantum Chemical Determination of Molecular Dye Candidates for Non-Invasive Bioimaging

**DOI:** 10.3390/molecules29245860

**Published:** 2024-12-12

**Authors:** Remy R. Cron, Jordan South, Ryan C. Fortenberry

**Affiliations:** 1 Department of Biochemistry and Molecular Genetics, University of Alabama-Birmingham, Birmingham, AL 35294, USA; rrcron@uab.edu; 2 Department of Chemistry & Biochemistry, University of Mississippi, University, MS 38677, USA; jlsouth1@go.olemiss.edu

**Keywords:** SWIR, π→π* photochemistry, density functional theory

## Abstract

Molecular dyes containing carbazole-based π bridges and/or julolidine-based donors should be promising molecules for intense SWIR emission with potential application to molecular bioimaging. This study stochastically analyzes the combinations of more than 250 organic dyes constructed within the D-π-D (or equivalently D-B-D) motif. These dyes are built from 22 donors (D) and 14 π bridges (B) and are computationally examined using density functional theory (DFT). The DFT computations provide optimized geometries from which the excited state transition wavelengths and associated oscillator strengths and orbital overlaps are computed. While absorption is used as a stand-in for emission, the longer the absorption wavelength, the longer the emission should be as well for molecules of this type. Nearly 100 novel dyes reported in this work have electronic absorptions at or beyond 1200 nm, opening the possibility for future synthesis and experimental characterization of new molecular dyes with promising properties for bioimaging.

## 1. Introduction

In vivo bioimaging promises to revolutionize non-destructive analysis of disease, tumors, or other biomarkers [1,2]. The relatively long wavelengths of electronic excitations exhibited in the short-wavelength infrared (SWIR) region from 1000 to 2000 nm allow organic dyes/fluorophores to penetrate body tissues so that they can provide imaging that does not require invasive or toxic techniques. Several organic dyes for this application already have approval from the United States Food and Drug Administration (FDA), like indocyanine green (ICG) [1,3], but they lack the clarity needed to resolve intricate details of the biological structure. Longer wavelength dyes, especially beyond the 1450 nm water absorption feature, will perform better as they do not interfere with the background signal from biological matrix fluorescence and scattering coefficients nearly as much [4], while the current longest-wavelength bioimaging dye emits at 1550 nm (SiRos1550) [5] and another at 1380 nm (NIR1380) [6]; other dyes have emission tails beyond 1600 nm (VIX-4) [7]. These dyes have promising properties for bioimaging, but many others may be possible that exhibit better behavior for such an application.

However, the synthesis and analysis of a single, novel molecular dye for application to bioimaging as well as the closely related field of solar energy harvesting can cost more than $10,000 even if the dye turns out to be suboptimal. While organic chemists working in this field have demonstrated almost preternatural intuition for finding exceptional structures in many cases [8,9,10,11], the inevitability is that other promising candidates, especially those of surprising chemical novelty, are undoubtedly missed. In order to alleviate such issues, computational chemistry can combinatorially screen a much more significant swathe of candidate molecules so that non-intuitive, synthetically challenging, or otherwise disregarded structures can be analyzed for the cheap cost of computer cycles [12,13,14]. Computational, combinatorial techniques can provide initial screening data for hundreds to thousands of dye candidates and generate novel molecules with promising, long-wavelength properties in fractions of the time and money of what exploratory, experimental characterization can do [15]. This promises to deliver new molecular dye candidates for later experimental analysis and, potentially, also to encourage the development of new synthetic approaches for novel dyes that would not otherwise be explored.

The organic molecules required for application to molecular bioimaging are not that different from those present in dye-sensisitized solar cells (DSCs). The light absorption properties of organic dyes for DSC applications have been improved over the years by altering the highest occupied molecular orbital (HOMO) and lowest unoccupied molecular orbital (LUMO) energies as a result of changing the structure of the organic dye molecule. A common means of constructing a dye molecule is incorporation of an electron donor (D), π-bridge (π), and electron acceptor (A) group each into a given organic molecule, creating the D-π-A molecular motif. The electron donor is an electron-rich moiety that donates electron density to the electron acceptor, and the energy of this donation is affected by the HOMO and LUMO energies [16]. Examples of common donor structures are triphenylamines and indolines [17]. The π-bridge structure is a π-conjugated system that transfers the electron from the donor to the acceptor upon photo excitation and assists with tuning the HOMO-LUMO energy gap. Examples of common π-bridge structures include benzothiadiazole, thiophene, and porphyrin [17]. The electron acceptor collects the electrons from the π-bridge upon excitation and is anchored to the semiconductor [18]. Examples of common electron acceptors include cyanoacrylic acid and benzoic acid [19]. Modifying the electron donor, π-bridge, and/or electron acceptor can improve the performance of DSCs by designing organic molecular dyes to maximize the desired properties. This same approach can be applied to other applications for molecules that required similar photophysical behavior.

In fact, a D-π-D motif is emerging for dyes needed in bioimaging; while this is similar to the D-π-A molecular motif for DSCs [15], the D-π-D (or equivalently D-B-D) construction is preferable in bioimaging dyes as high-intensity, long-wavelength electronic emission is preferred, while DSCs require charge-transfer to move the electron density from one side of the molecule to the other side and subsequently into the circuit, bioimaging dyes need to send as many, high intensity photons through the tissue as possible. Hence, strong orbital overlap and large oscillator strengths are required. Like in DSCs, HOMO-LUMO, $\pi \to \pi^{‗}$ excitations in the NIR/SWIR are preferred as they have the most promise for allowing molecular dye absorption and emission to be able to pass through human and animal tissue [20]. While these dyes sacrifice some of the long-wavelength emissions in charge-transfer systems similar in construction to the DSCs above [21], they more than make up for this by way of significantly improved molar absorptivity and quantum yield [20]. Such dyes often exhibit smaller widths of absorption/emission peaks and smaller Stokes shifts but much higher molar absorptivity [22]. Even though higher molar absorptivity does not always guarantee solid performance [7], several types of donors and π bridges are known to work well, including flavylium polymethine dyes [20]. However, more donors and π bridges are waiting to be explored, and quantum chemical computation once more promises the fastest throughput for initial screening of these molecules. As such, this work will explore several D-π-D (D-B-D) structures computationally and score them based on a combination of their high HOMO-LUMO overlap, large electronic transition intensity, and long excitation wavelength.

## 2. Results and Discussion

**Figure 1:**
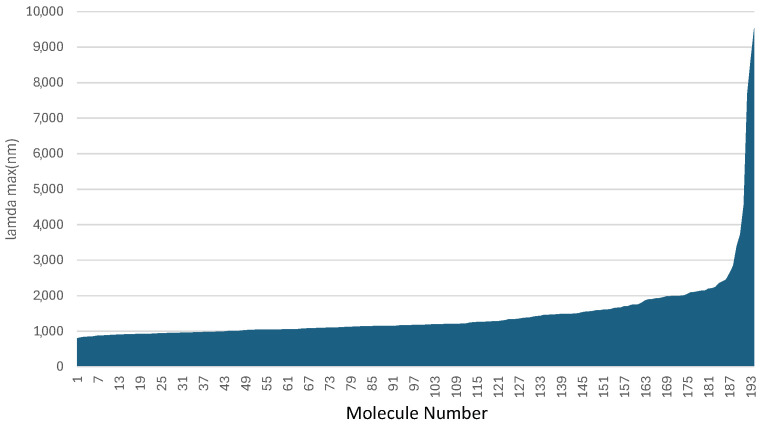
The wavelengths computed for the constructed D-π-D (D-B-D) dyes. The x-axis is largely arbitrary as the dyes are sorted by wavelength.

Arguably, the wavelength is the most important value to be computed. Without that, none of the other properties matter much. As such, the wavelengths for all of the molecules examined in this work are plotted in Figure 1. Several tested structures were found to not be stable or minima of the combinations of the D-B-D dyes. Hence, those are not included in this subsequent analysis. Regardless, Figure 1 highlights several molecules that have HOMO-LUMO transitions in the promising SWIR region. More than 20 have computed wavelengths of $\lambda_{max}$ weighted wavelengths of 2000 nm or more. Of course, those with much longer wavelengths should be taken with a grain of salt, again, as small changes to the excitation energy can influence the wavelength predicted. However, the wavelength has to be balanced by the oscillator strength and orbital overlap. As such, the total molecule must be evaluated for its possible role as a future dye and not just the wavelength.

**Figure 2:**
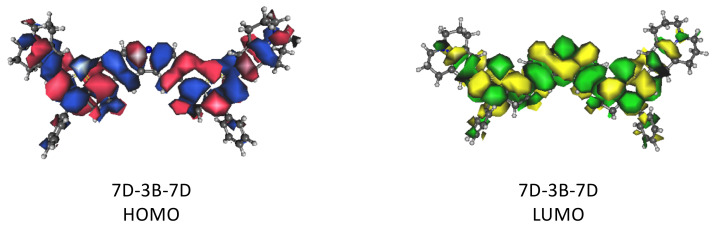
The orbitals involved in the 7D-3B-7D HOMO-LUMO transition.

The dye with the highest score, as shown in Table 1, is 7D-3B-7D. 7D is a julolidine-based donor akin to the donor in a promising DSC dye predicted and synthesized previously [15], and 3B is a carbazole-based π bridge also synthesized previously as part of other dyes for different dye applications [23]. This 7D-3B-7D molecular dye scores 232 with a $\lambda_{max}$ computed to be an impressive 2400 nm and HOMO-LUMO orbital overlap of 88%. However, the $f_{c}$ score is 44 for an $f_{avg}$ value between the two TD-DFT computations of only 1.329. While relatively low compared to many of the other dyes examined herein, this oscillator strength is still notably high in a general sense as most organic molecules do not exhibit *f* values for their electronic excitations of more than 1.0. The complete set of all values computed for creating these scores can be found in Table S1 of the Supplementary Materials. The orbital overlap for the 7D-3B-7D dye is depicted in Figure 2. Again, the overlap for this dyes is 88%. This is very high since the HOMO and LUMO are very nearly perfectly hosting electrons in the same volume. Even so, none of these three sub-scores and their underlying properties are the highest of the tested group, but their combination is high enough in the case of 7D-3B-7D to produce the largest score. Such a score should imply favorable photophysics for the long-wavelength HOMO-LUMO excitation in this molecular dye candidate.

**Figure 3:**
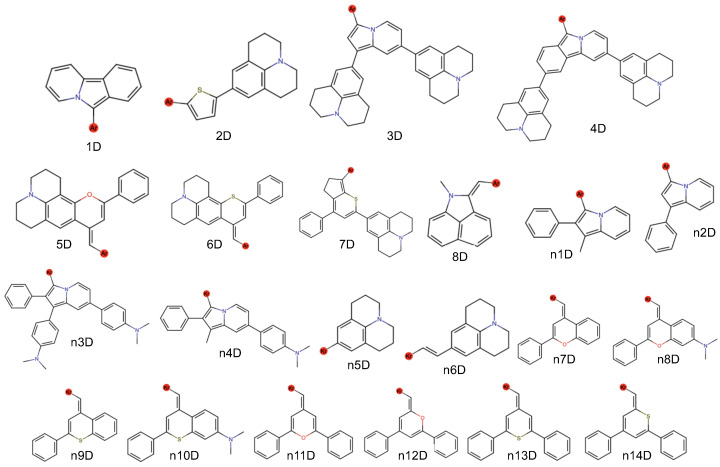
Chemical structures for the donors utilized in this work. Note the red noble gas (argon) atoms used as placeholders for where the linkages are made.

The next highest scoring dye is 4D-2B-4D with a score of 227, as shown in Table 1. This dye possesses another julolidine-based donor, but there are two such groups in the 4D donor fragment, unlike the lone julolidine in 7D. The 4D-2B-4D dye has $\lambda_{max}$ at 1984 nm, near to the maximum scoring wavelength of 2000 nm, and the emission should be even longer. The oscillator strength is nearly 2.0, and the orbital overlap is 65%. Hence, this molecule is also not the top performer in any category and is, frankly, not even close. However, the sum of the sub-scores together makes for a quality total score. The next two highest-scoring dyes come in at 223 and 221 each, respectively, of 3D-5B-3D and 4D-5B-4D. The 3D and 4D donor portions are both dijulolidine structures and differ only by an additional benzene right in the center of the structure (Figure 3). Consequently, the top performers from our tested set are those with julolidine termini.

While 7D-3B-7D has the longest wavelength of the highest-scoring dyes, the highest $f_{c}$ value is for 2D-n4B-2D. The n4B bridge is similar to the 5B bridge in the third- and fourth-highest scoring dyes, but it has a phosphorus in the central ring instead of a sulfur. Regardless, the wavelength is fairly short, leading to a total score of “only” 161 even though $f_{c}$ is 93 with $f_{avg}$ equal to a whopping 2.826.

The highest orbital overlap is for n14D-3B-n14D at 91%, putting this dye in the top 10 with a score of 211. Again, the high overlap is present from the inclusion of 3B in the dye structure. The relatively simple 3B, carbazole-based π bridge, in general, produces the highest orbital overlaps for all of the examined dyes, as shown in Table 1. Consequently, 3B appears to be the best-performing π bridge fragment in this D-B-D motif.

The total set of all data and scores for the total 259 molecular dyes explored for this bioimaging application are given in the Supplementary Materials. A toptal of 170 of the dye candidates have $\lambda_{max}$ values below 1200 nm and, hence, total scores of 0 by definition. Only six scores between 0 and 100, and 68 scores between 100 and 200. Those 15 molecular dye candidates with scores of more than 200 are, again, given in Table 1 along with two others retained therein for discussion. These numbers show that a majority of the dyes examined in this work would not be viable candidates for synthesis. While most experimental chemists working in this area would probably be able to rationalize a similar conclusion that these dyes are not viable, conclusive evidence is now given in order to guide future synthesis. While many of these dyes are promising, only three criteria have been considered. The present work is limited in not being able to predict toxicity or synthetic ease, but of the 259 examined, 83 have varying levels of viability as bioimaging molecular dyes, implying that hundreds of candidates have now become tens. Not all of the final set would be desirable targets, not only for the scores given here. Hence, synthetic chemists can examine the list from Table 1 and from the Supplementary Materials to determine which would be fitting to examine and create.

In terms of synthesis, a promising candidate is likely 8D-3B-8D from consultation with synthetic chemists (see Acknowledgments). While this molecular dye candidate does exhibit a long, 1932 nm excitation wavelength and 85% overlap, its oscillator strength is only 0.647. This is quite low, leading to a score of 197. However, the strong overlap could still lead to a high quantum yield. From a synthetic perspective, this molecule is desirable mainly for its π bridge. The carbazole π bridge present in 3B is a common organic molecular scaffold and has established techniques for synthesis and functionalization thereon [23]. Additionally, the 8D groups are not much of a synthetic to work with as some of the other donors would be. Many of the other carbazole-based dyes with a 3B π bridge are promising as well, but their donor groups have not been as commonly utilized in the literature. Again, we leave the final assessment of what dyes to examine to the synthetic chemists based on their expertise and limitations, but this list should provide those with the expertise to create and test such molecular dye candidates with promising targets for future characterization.

## 3. Computational Methodology

### 3.1. Dye Enumeration & Quantum Chemical Computations

**Figure 4:**
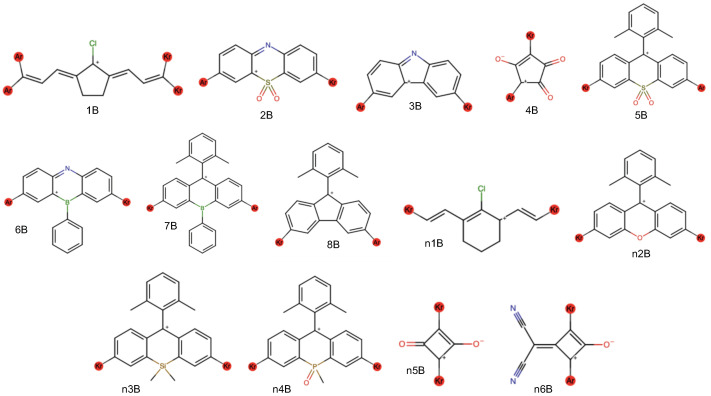
Chemical structures for the π bridges utilized in this work with red noble gas atom placeholders. Since there are two connection sites in these molecules, different noble gas atom placeholder (argon & krypton) are used.

The computational procedure is similar to that done recently for DSC applications [15]. The first step requires a list of potential donor (D) and π bridge (B) fragments, numbered in Figure 3 and Figure 4, respectively, to first be generated based upon the literature and chemical intuition. After an initial run of fragments, additional donor and bridge fragments have also been tested leading to the “n” notation for the fragment names. This produces a total of 22 donors and 14 π bridges. However, the 1B fragment has been omitted as its four linkages produce a large number of potential dyes that are likely difficult to synthesize as described by consultation with organic chemists (see Acknowledgements). The π bridges (B) are all cations to create an overall positively charged dye. This is necessary to create the salt for stable storage and subsequent dissolving into solution.

Regardless, for the computational procedure, the structure for each of these fragments is converted into a SMILES string and put into a .smi file. The connections to the donor and backbone fragments are represented as krypton atoms in these SMILES strings. The in-house written Python script enumerate_dyes.py utilizes input .smi files and first converts them to molecular graphs. Then, the fragments are combined in all possible combinations of donors and backbones to generate molecular graphs of the dye candidates. These molecular graphs are then converted into SMILES strings once more and subsequently into InchI strings. The InchI strings, which are unique to each molecule, are then compared against one another and duplicate dye candidates are excluded from subsequent analysis.

The in-house generate_jobs.py script runs the enumerate_dyes.py script, creating a SMILES string for each of the dye candidates. The generate_jobs.py script then creates an initial guess of the Cartesian coordinates of each of the dye candidates using OpenBabel [24]. The Cartesian coordinates are used to generate a single, compound Gaussian16 [25] input file where multiple job types are run along with producing a submission script for each of the candidate dyes. Each dye candidate requires the following computations: (1) a geometry optimization using the B3LYP/6-311G(d,p) level of theory [26,27,28,29], (2) a single excited state calculation using the CAM-B3LYP/6-311G(d,p) level of theory [30], (3) a second excited state calculation using the PBE0/6-311G(d,p) level of theory [31,32], and (4) a molecular orbital calculation using the PBE0/6-311G(d,p) level of theory. This is very similar to the approach previously employed for DSCs [15], while all of the possible combinations of D and B connections are created within the D-π-D (or equivalently D-B-D) motifs, only those with the same D on either side of the B have been computed herein as these make the most reasonable synthetic targets.

### 3.2. Scoring

The extract_scores.py script is used to score each dye candidate using the newly computed data from its corresponding Gaussian output file. Equations for the total score, *s* (Equation (1)):

(1) $$s=l+f_{c}+o,$$

and its sub-scores *l*, $f_{c}$, and *o* are shown in Equations (3), (5) and (6). The *l* score is for maximizing the wavelength as close to 2000 nm as possible. Each of the sub-scores *l*, $f_{c}$, and *o* are intended to take on values from 0 to 100 for most dye candidates. This design constrains the total score *s* from 0 to 300 in most cases, with a score of 300 representing a top performing biomedical imaging dye. This scoring procedure is developed in this work in order to provide a numerical estimate for the performance of the dye. A discussion of each portion of the score and why the numbers are selected is given below.

The *l* sub-score represents how preferable a dye candidate is based on its estimated maximum absorption wavelength in nm, $\lambda_{max}$:

(2) $$\lambda_{max}=\frac{hc}{\Delta E_{lsf}}=\frac{hc}{0.36*\Delta E_{\mathrm{CAM}-B3\mathrm{LYP}}+0.58*\Delta E_{\mathrm{PBE}0}-0.31}.$$

$\lambda_{max}$ acts as a proxy for a dye candidate’s maximum emission wavelength, which is more challenging to compute en masse directly but is generally longer than a dye candidate’s maximum absorption wavelength. Hence, the longer the $\lambda_{max}$, the longer the emission wavelength is assumed to be as part of this screening protocol. $\lambda_{max}$ is calculated, first, from an estimate of the HOMO-LUMO transition energy in eV extracted from the single excited state CAM-B3LYP/6-311G(d,p) calculation. A similar estimate is extracted from the corresponding PBE0/6-311G(d,p) calculation. Next, an approximation of the transition energy, called $\Delta E_{lsf}$, is made using a least-squares fit model. This model is obtained using experimental data of dyes with known maximum absorbance wavelengths, which are used to compute their transition energies; the least-squares fit predicts the experimental transition energy as a function of the approximate transition energies computed using CAM-B3LYP/6-311G(d,p) and PBE0/6-311G(d,p) and has been determined from previous work on DSC molecular dyes where the mean absolute error compared to experiment is 0.13 eV [15]. Additional comparison to unpublished experimental dye spectra suggests that this error could even be as low as 0.10 eV. Finally, $\lambda_{max}$ is determined from $\Delta E_{lsf}$ using the Planck–Einstein relation and converted into wavelengths in nm.

The *l* sub-score (Equation (3)) is a linear function of $\lambda_{max}$ with coefficients chosen such that a dye candidate with a 2000 nm or longer excitation wavelength is assigned a maximum score of 100, while a dye candidate with a 1200 nm or less excitation wavelength is assigned a minimum score of 0.

(3) $$l=100-\frac{\left(2000-\lambda_{max}\right)}{8},$$

All wavelengths below 1200 nm are automatically assigned not just an *l* score of 0 but a total *s* score of 0, as no other properties will be valuable for a dye candidate. Beyond 2000 nm, small errors in the excitation energy will lead to large shifts in the wavelength motivating the choice for 2000 nm as the maximum. Any dye candidates with wavelengths computed beyond this range are all scored the same so as not to bias the results in this way. The “8” denominator in the $\lambda_{max}$ computation defines 0 to be 1200 nm in this equation. Previous work [15] has shown that CAM-B3LYP/6-311G(d,p) tends to overestimate the excitation energies of dye-like molecules, while PBE0/6-311G(d,p) tends to underestimate the excitation energies of dye-like molecules. This is not perfectly linear and explains in part the denominator term in $\lambda_{max}$ in Equation (2).

The $f_{c}$ sub-score represents how preferable a dye candidate is based on its oscillator strength, a proxy for the intensity of the photophysical processes.

(4) $$f_{avg}=\frac{f(B3\mathrm{LYP})+f(\mathrm{PBE}0)}{2}$$

(5) $$f_{c}=100-\left[\left(3-f_{avg}\right)\times \frac{100}{3}\right]$$

The oscillator strength, $f_{avg}$, is calculated as a standard average of the oscillator strengths from the CAM-B3LYP/6-311G(d,p) excited state and PEB0/6-311G(d,p) simulations. Experimental data of oscillator strengths of dyes related to those being simulated were unavailable. Thus, an average of the results of the two density functions was used to obtain a potentially more accurate estimate of the oscillator strength than the estimates using either density functional alone. The $f_{c}$ sub-score is a linear function of *f* and has a perfect score of 100 when *f* is 3 and a failing score of 0 when *f* is 0. The choice of 3 as a maximum is selected through testing of the examining dyes and represents approximately the largest value computed. Again, the assumption is made that a high $f_{c}$ in absorption should correlate to a high $f_{c}$ in emission.

The *o* sub-score represents how preferable a dye candidate is based on the overlap between its HOMO and LUMO, a desirable property for these bioimaging dyes since only these orbitals are involved in the longest wavelength transitions.

(6) $$o=100*\sum_{i\in fragments}min\left({\mathrm{HOMO}}_{i},{\mathrm{LUMO}}_{i}\right)$$

This value is computed from the contribution of the atomic orbitals (AOs) to the molecular orbitals (MOs) in a standard LCAO-MO orbital construction utilized here. Each atom is defined to originate from a certain fragment of the molecule based on the above construction process. For the given MO (HOMO or LUMO), all of the AO volume contributions for an individual atom in the fragment are summed. Then, the AO contributions for each atom in the fragment are summed. This provides the total MO contribution from each fragment and is done for both the HOMO and the LUMO. Next, the volume overlap between the HOMO and the LUMO is computed as the minimum of these two volumes. This represents the amount of normalized electron density overlap between the HOMO and LUMO in that fragment. To obtain the total electron density overlap between these two orbitals, the overlaps over all fragments are summed, yielding a value in the range [0, 1]. This overlap value is multiplied by 100 to yield the *o* sub-score, which is in the range [0, 100]. This is effectively the same procedure as that done in ref. [15], but the scoring is inverted here as strong overlap is most important for a bioimaging dye as opposed to charge transfer in a DSC molecular dye. Regardless of absorption or emission, strong orbital overlap is key for promising photophysical behavior.

## 4. Conclusions

The 259 dye candidates examined here for application to in vivo bioimaging showcase that the carbazole-based π bridge 3B fragment typically produces high HOMO-LUMO overlap in the first electronically excited state for molecular dye candidates containing this fragment. This overlap can help to enhance emission strength and subsequent quantum yield of the dyes in question. The overlap consists of only one scoring component, with the other two including maximizing both the wavelength excitation energies and oscillator strengths. Additionally, of the 15 highest-scoring dyes, eight contain 3B, also showing that this π bridge contributes to long-wavelength excitation as well. In fact, the most common π bridge in the 83 dye candidates scoring above 100 in this work is 3B. Hence, this bridge is the most potent for the properties desired in this bioimaging application. The 8B along, with the 5B and related n4B π bridges, also provide for a number of the dyes that exhibit excitation wavelengths beyond 1200 nm, many close to 2000 nm. The recurrence of these four π bridges highlights that this portion of the dye is likely more influential than the donor groups on the performance of the dye candidate. That being said, the julolidine donors (2D–7D) can contribute to long excitation energy wavelengths, especially in conjunction with 3B. Such long-wavelength absorption should, by extension, imply longer-wavelength emission, as well.

Beyond these highlighted fragments, 83 dye candidates are computed in this work to exhibit excitation energies of more than 1200 nm, implying that their emission should be beyond even this level. Hence, any of them could be viable candidates for bioimaging dyes. The toxicity, biocompatibility, and synthetic accessibility of these dyes have yet to be seen and are left for future work. Even so, the present results are providing an initial computationally-derived catalogue for possible molecular dyes from which those properties can be determined and inspire future studies in this area.

## Figures and Tables

**Table 1:** Compiled, selected data for the most promising dyes computed in this work. The full data are given in the Supplementary Materials.

Dye	$\lambda_{\max}$	*l*	Avg.	$f_{c}$	*o*	Total
Name	(nm)	Score	f	Score	Score	Score
7D-3B-7D	2400	100	1.329	44	88	232
4D-2B-4D	1984	98	1.950	64	65	227
3D-5B-3D	1997	100	2.022	67	57	223
4D-5B-4D	1998	100	1.776	59	63	221
6D-6B-6D	1805	76	1.550	51	86	213
6D-3B-6D	2210	100	0.662	22	90	212
5D-3B-5D	2099	100	0.664	22	90	212
n13D-3B-n13D	1995	99	0.687	23	90	212
n14D-3B-n14D	1980	98	0.682	22	91	211
n3D-2B-n3D	1926	91	1.819	60	58	209
4D-8B-4D	2245	100	1.110	37	65	202
n11D-3B-n11D	2127	100	0.370	12	89	202
3D-n4B-3D	1870	84	1.910	63	54	201
n7D-3B-n7D	2048	100	0.411	14	87	201
n9D-3B-n9D	2148	100	0.403	13	87	200
8D-3B-8D	1932	92	0.647	21	85	197
2D-n4B-2D	1355	19	2.826	93	48	161

## Data Availability

Data needed in support of this research can be found in the Supplementary Materials.

## Supplementary Materials

The following supporting information can be downloaded at: https://www.mdpi.com/article/10.3390/molecules29245860/s1. The raw data for the dyes examined in this work.

## Abbreviations

The following abbreviations are used in this manuscript:

DFT	Density Functional Theory
SWIR	Short Wavelength Infrared
HOMO	Highest Occupied Molecular Orbital
LUMO	Lowest Unoccupied Molecular Orbital
DSC	Dye Sensistized Solar Cell
D-π-D	Donor-Pi-Donor
D-B-D	Donor-Backbone-Donor
FDA	Food and Drug Administration (U.S.A.)
LCAO-MO	Linear Combination of Atomic Orbitals to make Molecular Orbitals
