# Supplementary material for: Quantum Chemical Determination of Molecular Dye Candidates for Non-Invasive Bioimaging
Source: Molecules. 2024 Dec 12;29(24):5860. doi: 10.3390/molecules29245860 (PMC11677789; doi:10.3390/molecules29245860)
Supplement: Supplementary file 1 [file molecules-29-05860-s001.zip › molecules-3337978-supplementary.pdf]

Supporting Information for: Quantum Chemical Determination of Molecular Dye Candidates for Non-Invasive Bioimaging

Remy N. Cron,[1,2] Jordan South,[1] and Ryan C. Fortenberry*,[1]

1*Department of Chemistry & Biochemistry, University of Mississippi, University, MS 38677, USA*

2*Department of Biochemistry and Molecular Genetics, University of Alabama-Birmingham, Birmingham, AL 35294, USA*

E-mail: r410@olemiss.edu

Table S1: The raw data for the dyes examined in this work. Note that the ordering in the name is changed from D-B-D to the equivalent D-D-B. A single "n" on the donor name should be understood to have the "n" on both D portions. Those beginning with "nnn" at the beginning should be understood to have "n" before both D and the B portions.

Dye Name	CAM-B3LYP XS (eV)	PBE0 XS (eV)	LSF XS (eV)	λ_{max} (nm)	l Score	CAM-B3LYP f	PBE0 f	Avg. f	f_c Score	o Score	Total Score
7D-7D-3B	0.833	0.909	0.517	2400	100	1.359	1.300	1.329	44	88	232
4D-4D-2B	1.103	0.927	0.625	1984	98	2.566	1.334	1.950	64	65	227
3D-3D-5B	1.088	0.930	0.621	1997	100	2.454	1.590	2.022	67	57	223
4D-4D-5B	1.083	0.932	0.620	1998	100	2.057	1.496	1.776	59	63	221
6D-6D-6B	1.022	1.084	0.687	1805	76	1.665	1.435	1.550	51	86	213
6D-6D-3B	0.867	0.964	0.561	2210	100	0.695	0.630	0.662	22	90	212
5D-5D-3B	0.903	0.992	0.591	2099	100	0.693	0.634	0.664	22	90	212
n13D-13D-3B	0.931	1.028	0.621	1995	99	0.725	0.650	0.687	23	90	212
n14D-14D-3B	0.938	1.032	0.626	1980	98	0.714	0.649	0.682	22	91	211
n3D-3D-2B	1.123	0.947	0.644	1926	91	2.270	1.369	1.819	60	58	209
4D-4D-8B	0.964	0.888	0.552	2245	100	1.274	0.946	1.110	37	65	202
n11D-11D-3B	0.894	0.985	0.583	2127	100	0.386	0.353	0.370	12	89	202
3D-3D-n4B	1.145	0.967	0.663	1870	84	2.222	1.598	1.910	63	54	201
n7D-7D-3B	0.912	1.012	0.605	2048	100	0.426	0.396	0.411	14	87	201
n9D-9D-3B	0.879	0.984	0.577	2148	100	0.419	0.387	0.403	13	87	200
n4D-4D-3B	0.832	0.890	0.506	2453	100	0.755	0.764	0.759	25	74	199
8D-8D-3B	0.965	1.042	0.642	1932	92	0.671	0.624	0.647	21	85	197
1D-1D-3B	0.936	1.017	0.617	2010	100	0.538	0.469	0.503	17	80	196
5D-5D-6B	1.050	1.101	0.706	1755	69	1.366	1.172	1.269	42	84	195
n12D-12D-3B	0.979	1.063	0.659	1881	85	0.669	0.613	0.641	21	89	195
n3D-3D-3B	0.699	0.731	0.365	3393	100	0.618	0.620	0.619	20	73	193
3D-3D-8B	0.948	0.856	0.528	2350	100	1.078	0.929	1.003	33	60	193
7D-7D-8B	1.022	1.025	0.652	1902	88	0.866	0.808	0.837	28	75	190
n1D-1D-3B	0.897	0.977	0.580	2139	100	0.379	0.327	0.353	12	76	188
n10D-10D-3B	0.884	0.956	0.563	2203	100	0.357	0.340	0.349	12	75	186
n2D-2D-3B	0.966	1.031	0.635	1951	94	0.584	0.540	0.562	19	74	186
4D-4D-n3B	1.219	1.030	0.726	1707	63	2.403	1.637	2.020	67	56	186
3D-3D-n3B	1.253	1.012	0.728	1703	63	2.596	1.740	2.168	72	51	185
3D-3D-n1B	1.477	1.300	0.975	1271	9	3.127	2.277	2.702	89	76	174
6D-6D-8B	1.126	1.163	0.770	1610	51	1.285	1.223	1.254	41	80	173
2D-2D-5B	1.307	1.244	0.882	1405	26	2.929	2.778	2.853	94	50	170
n4D-4D-8B	1.114	1.069	0.711	1744	68	1.234	1.369	1.302	43	58	169
2D-2D-6B	0.687	0.681	0.332	3731	100	0.632	0.775	0.704	23	43	166
7D-7D-2B	1.135	1.112	0.744	1667	58	1.061	0.786	0.924	30	76	165
1D-1D-6B	1.097	1.149	0.751	1651	56	1.108	0.941	1.025	34	74	164
8D-8D-6B	1.123	1.158	0.766	1618	52	1.076	0.953	1.015	33	78	163
n4D-4D-5B	1.297	1.196	0.851	1458	32	2.366	2.231	2.298	76	55	163
3D-3D-2B	1.013	0.716	0.470	2639	100	0.959	0.872	0.915	30	32	163
2D-2D-n4B	1.366	1.264	0.915	1355	19	2.910	2.743	2.826	93	48	161
2D-2D-2B	1.164	0.939	0.654	1897	87	1.328	1.105	1.217	40	32	160
3D-3D-6B	0.493	0.476	0.144	8619	100	0.333	0.515	0.424	14	45	159
n10D-10D-2B	1.403	1.434	1.027	1207	1	2.421	2.286	2.354	78	79	158
7D-7D-5B	1.149	1.109	0.747	1660	58	0.999	0.732	0.865	29	71	157
4D-4D-n1B	1.558	1.348	1.032	1201	0	2.859	1.798	2.328	77	80	157
6D-6D-5B	1.352	1.370	0.972	1276	10	2.224	2.047	2.136	70	76	156
4D-4D-3B	0.703	0.323	0.130	9536	100	0.761	0.071	0.416	14	36	150

5D-5D-8B	1.159	1.189	0.797	1556	44	0.798	0.770	0.784	26	79	149
5D-5D-5B	1.421	1.422	1.026	1208	1	2.281	2.087	2.184	72	76	149
2D-2D-n3B	1.467	1.290	0.966	1283	10	2.908	2.683	2.796	92	45	148
4D-4D-n2B	1.407	1.109	0.840	1477	35	2.313	1.293	1.803	59	52	146
6D-6D-2B	1.349	1.365	0.967	1282	10	1.832	1.669	1.751	58	78	146
2D-2D-8B	1.108	1.068	0.708	1751	69	0.721	0.715	0.718	24	53	146
n4D-4D-2B	1.237	1.147	0.801	1549	44	1.502	1.223	1.363	45	57	146
n6D-6D-5B	1.436	1.412	1.026	1208	1	2.771	2.651	2.711	89	54	144
6D-6D-4B	1.305	1.372	0.955	1298	12	1.199	1.083	1.141	38	92	142
4D-4D-4B	1.361	1.384	0.983	1262	8	1.385	1.341	1.363	45	89	141
n14D-14D-4B	1.386	1.436	1.022	1214	2	1.476	1.286	1.381	46	93	141
5D-5D-2B	1.415	1.415	1.020	1215	2	1.899	1.712	1.806	60	78	139
n14D-14D-8B	1.144	1.179	0.786	1578	47	0.409	0.399	0.404	13	78	139
3D-3D-3B	0.640	0.416	0.162	7677	100	0.103	0.211	0.157	5	33	139
n13D-13D-8B	1.135	1.174	0.780	1590	49	0.398	0.388	0.393	13	76	138
n10D-10D-8B	1.148	1.152	0.772	1607	51	0.406	0.394	0.400	13	72	136
2D-2D-3B	0.821	0.494	0.272	4556	100	0.165	0.275	0.220	7	27	135
n6D-6D-8B	1.215	1.221	0.835	1484	36	1.194	1.215	1.205	40	59	134
nnn4D-4D-3B	1.423	1.185	0.889	1394	24	1.961	1.561	1.761	58	51	133
n6D-6D-3B	0.914	0.523	0.323	3843	100	0.128	0.173	0.150	5	28	133
5D-5D-4B	1.391	1.438	1.025	1209	1	1.291	1.179	1.235	41	90	132
n9D-9D-8B	1.170	1.192	0.803	1545	43	0.442	0.442	0.442	15	72	130
3D-3D-n2B	1.433	1.082	0.833	1488	36	1.596	1.157	1.376	45	48	130
n7D-7D-8B	1.180	1.167	0.792	1566	46	0.402	0.382	0.392	13	71	130
5D-5D-n4B	1.441	1.419	1.032	1201	0	1.823	1.632	1.727	57	72	129
n11D-11D-8B	1.269	1.295	0.898	1381	23	0.808	0.788	0.798	26	80	128
n5D-5D-3B	1.173	0.559	0.437	2840	100	0.065	0.078	0.072	2	25	127
8D-8D-8B	1.250	1.256	0.868	1428	28	0.774	0.779	0.777	26	71	125
n12D-12D-8B	1.199	1.222	0.830	1493	37	0.397	0.392	0.395	13	75	125
1D-1D-8B	1.201	1.229	0.835	1484	36	0.628	0.593	0.610	20	66	121
n2D-2D-8B	1.199	1.182	0.807	1536	42	0.560	0.578	0.569	19	59	120
n10D-10D-5B	1.371	1.388	0.988	1255	7	1.182	1.075	1.128	37	74	118
nnn10D-10D-4B	1.405	1.403	1.010	1228	4	1.251	1.135	1.193	39	72	115
n9D-9D-4B	1.355	1.386	0.981	1263	8	1.346	0.000	0.673	22	82	113
nnn3D-3D-2B	1.500	1.127	0.883	1404	25	1.356	0.897	1.126	37	44	106
n1D-1D-8B	1.283	1.284	0.896	1383	23	0.616	0.592	0.604	20	60	103
7D-7D-6B	1.836	0.411	0.589	2105	100	0.043	0.020	0.031	1	1	103
nnn9D-10D-6B	1.442	1.382	1.011	1227	3	0.806	0.630	0.718	24	72	99
nnn10D-10D-3B	1.564	1.341	1.030	1203	0	1.705	0.313	1.009	33	62	96
n5D-5D-8B	1.333	1.311	0.930	1333	17	0.658	0.687	0.673	22	55	94
nnn9D-9D-3B	1.650	1.263	1.017	1220	2	1.654	0.065	0.860	28	62	93
n5D-5D-2B	1.468	1.197	0.913	1359	20	1.039	0.741	0.890	29	37	87
1D-1D-3B	1.629	1.246	0.999	1242	5	1.000	0.080	0.540	18	58	81
n6D-6D-2B	1.433	1.428	1.034	1199	0	2.726	2.603	2.664	88	58	0
nnn9D-9D-6B	1.476	1.405	1.036	1196	0	0.971	0.686	0.829	27	80	0
nnn4D-4D-2B	1.616	1.320	1.037	1195	0	2.071	1.770	1.921	63	45	0
n10D-10D-4B	1.327	1.501	1.038	1194	0	1.369	0.002	0.686	23	83	0
3D-3D-4B	1.452	1.425	1.039	1193	0	1.604	1.480	1.542	51	92	0
n14D-14D-2B	1.422	1.451	1.044	1188	0	1.399	1.328	1.364	45	79	0
n13D-13D-2B	1.424	1.464	1.052	1179	0	1.426	1.370	1.398	46	77	0
nnn10D-10D-6B	1.487	1.429	1.054	1176	0	1.065	0.816	0.941	31	81	0
n2D-2D-2B	1.491	1.427	1.054	1176	0	1.740	1.492	1.616	53	61	0
n7D-7D-4B	1.423	1.470	1.055	1176	0	1.390	1.235	1.313	43	79	0
n13D-13D-4B	1.398	1.486	1.055	1175	0	1.423	1.322	1.372	45	97	0
5D-5D-n6B	1.495	1.431	1.058	1172	0	0.941	0.758	0.850	28	71	0

nnn7D-7D-3B	1.708	1.303	1.060	1169	0	1.741	0.063	0.902	30	69	0
1D-1D-n3B	1.572	1.388	1.061	1168	0	1.232	0.194	0.713	24	57	0
nnn6D-6D-4B	1.494	1.440	1.063	1167	0	2.802	2.679	2.741	90	52	0
1D-1D-5B	1.459	1.466	1.065	1164	0	1.518	1.407	1.462	48	63	0
1D-1D-2B	1.450	1.471	1.065	1164	0	1.549	1.448	1.498	49	67	0
n9D-9D-2B	1.451	1.483	1.073	1156	0	2.417	2.328	2.372	78	75	0
n9D-9D-5B	1.459	1.479	1.073	1155	0	2.322	2.248	2.285	75	72	0
nnn7D-7D-6B	1.515	1.445	1.073	1155	0	0.840	0.674	0.757	25	67	0
2D-2D-n2B	1.660	1.357	1.075	1153	0	1.680	1.398	1.539	51	40	0
nnn14D-10D-6B	1.521	1.444	1.075	1153	0	1.083	0.799	0.941	31	77	0
nnn14D-14D-6B	1.538	1.439	1.078	1150	0	0.901	0.621	0.761	25	81	0
n1D-1D-2B	1.485	1.477	1.081	1147	0	1.326	1.194	1.260	42	59	0
1D-1D-n4B	1.488	1.475	1.081	1147	0	1.580	1.443	1.512	50	60	0
n2D-2D-5B	1.535	1.449	1.083	1145	0	1.735	1.444	1.589	52	56	0
2D-2D-n1B	1.515	1.464	1.085	1143	0	3.717	3.573	3.645	120	71	0
n14D-14D-5B	1.475	1.490	1.085	1143	0	1.986	1.908	1.947	64	76	0
nnn1D-10D-6B	1.564	1.437	1.086	1141	0	0.580	0.429	0.504	17	61	0
nnn2D-6D-4B	1.538	1.457	1.089	1139	0	2.289	1.931	2.110	70	53	0
n4D-4D-4B	1.490	1.490	1.091	1137	0	1.165	0.965	1.065	35	88	0
nnn13D-13D-6B	1.531	1.466	1.091	1136	0	0.982	0.692	0.837	28	85	0
8D-8D-5B	1.512	1.484	1.095	1132	0	1.893	1.825	1.859	61	67	0
5D-5D-n3B	1.525	1.479	1.097	1131	0	2.428	2.225	2.326	77	71	0
nnn9D-9D-4B	1.499	1.495	1.097	1130	0	1.978	1.912	1.945	64	69	0
nnn3D-3D-1B	1.607	1.433	1.100	1127	0	2.405	1.860	2.133	70	75	0
n7D-7D-5B	1.509	1.499	1.103	1124	0	1.993	1.919	1.956	65	73	0
8D-8D-2B	1.516	1.503	1.107	1120	0	2.026	1.956	1.991	66	72	0
nnn13D-7D-6B	1.561	1.475	1.108	1119	0	1.079	0.766	0.922	30	71	0
n12D-12D-4B	1.504	1.517	1.111	1116	0	1.339	1.154	1.246	41	93	0
nnn14D-14D-4B	1.516	1.516	1.115	1112	0	2.076	1.994	2.035	67	75	0
nnn12D-10D-6B	1.587	1.478	1.119	1108	0	1.063	0.769	0.916	30	74	0
n12D-12D-2B	1.515	1.524	1.120	1107	0	1.445	1.379	1.412	47	75	0
n1D-1D-5B	1.539	1.512	1.121	1106	0	1.473	1.324	1.399	46	56	0
n13D-13D-5B	1.507	1.533	1.122	1105	0	2.611	2.522	2.566	85	74	0
nnn6D-6D-3B	1.594	1.480	1.122	1105	0	2.877	2.726	2.801	92	49	0
nnn2D-2D-4B	1.589	1.488	1.125	1102	0	1.771	1.487	1.629	54	54	0
nnn13D-13D-4B	1.524	1.535	1.129	1098	0	2.156	2.080	2.118	70	73	0
n7D-7D-2B	1.520	1.540	1.131	1097	0	2.484	2.412	2.448	81	75	0
nnn3D-3D-6B	1.588	1.500	1.132	1096	0	1.010	0.814	0.912	30	91	0
nnn12D-12D-4B	1.545	1.528	1.132	1095	0	1.302	1.243	1.272	42	69	0
nnn1D-1D-4B	1.568	1.515	1.133	1094	0	1.472	1.324	1.398	46	54	0
8D-8D-n4B	1.569	1.523	1.138	1090	0	2.350	2.268	2.309	76	67	0
n11D-11D-4B	1.509	1.570	1.144	1084	0	1.633	1.528	1.580	52	93	0
nnn10D-6D-6B	1.608	1.517	1.149	1079	0	1.183	0.902	1.042	34	76	0
nnn10D-10D-1B	1.558	1.556	1.153	1075	0	1.900	1.796	1.848	61	94	0
n12D-12D-5B	1.560	1.557	1.154	1074	0	1.964	1.887	1.926	64	72	0
nnn2D-5D-4B	1.621	1.521	1.156	1073	0	1.810	1.559	1.684	56	50	0
nnn14D-14D-3B	1.595	1.559	1.168	1061	0	2.198	2.111	2.154	71	72	0
nnn9D-9D-1B	1.565	1.583	1.172	1058	0	1.980	1.929	1.955	65	95	0
8D-8D-n6B	1.635	1.540	1.172	1058	0	1.167	0.883	1.025	34	76	0
n5D-5D-5B	1.614	1.553	1.172	1058	0	1.875	1.775	1.825	60	49	0
5D-5D-n1B	1.600	1.570	1.177	1054	0	1.890	1.751	1.821	60	92	0
n11D-11D-2B	1.573	1.591	1.179	1051	0	2.295	2.230	2.263	75	79	0
2D-2D-4B	1.577	1.590	1.180	1051	0	2.132	2.224	2.178	72	86	0
nnn12D-12D-6B	1.681	1.526	1.180	1051	0	0.906	0.616	0.761	25	77	0
nnn2D-2D-3B	1.672	1.531	1.180	1051	0	1.808	1.537	1.672	55	51	0

8D-8D-n3B	1.638	1.554	1.181	1050	0	2.073	1.988	2.030	67	64	0
nnn1D-12D-6B	1.707	1.522	1.187	1044	0	0.718	0.475	0.596	20	56	0
nnn13D-13D-3B	1.610	1.584	1.188	1043	0	2.306	2.223	2.265	75	70	0
n11D-11D-5B	1.592	1.599	1.191	1041	0	2.570	2.509	2.539	84	75	0
1D-1D-4B	1.579	1.608	1.191	1041	0	0.863	0.619	0.741	24	95	0
nnn10D-10D-5B	1.589	1.602	1.191	1041	0	1.412	1.258	1.335	44	93	0
8D-8D-4B	1.445	1.696	1.194	1038	0	1.380	0.000	0.690	23	81	0
4D-4D-n6B	1.641	1.578	1.196	1037	0	1.456	1.360	1.408	46	92	0
nnn4D-4D-1B	1.660	1.586	1.207	1027	0	2.789	2.714	2.752	91	80	0
nnn12D-6D-6B	1.731	1.543	1.208	1027	0	1.170	0.775	0.972	32	63	0
nnn5D-5D-4B	1.675	1.584	1.211	1023	0	1.899	1.797	1.848	61	47	0
n1D-1D-4B	1.576	1.649	1.214	1022	0	0.718	0.566	0.642	21	89	0
nnn14D-14D-5B	1.656	1.612	1.221	1015	0	1.159	0.956	1.057	35	93	0
nnn2D-14D-6B	1.699	1.586	1.221	1015	0	0.940	0.653	0.796	26	70	0
nnn11D-11D-4B	1.635	1.626	1.222	1015	0	2.692	2.628	2.660	88	74	0
nnn12D-10D-1B	1.656	1.617	1.224	1013	0	2.069	1.910	1.989	66	86	0
n6D-6D-4B	1.892	1.484	1.232	1006	0	0.000	0.000	0.000	0	96	0
2D-2D-n6B	1.706	1.607	1.236	1003	0	1.620	1.385	1.503	50	99	0
5D-5D-n5B	1.654	1.640	1.237	1002	0	1.507	1.372	1.440	48	95	0
nnn14D-13D-5B	1.661	1.642	1.241	999	0	1.233	1.028	1.131	37	90	0
nnn12D-12D-3B	1.683	1.631	1.242	999	0	2.163	2.072	2.117	70	70	0
nnn11D-11D-6B	1.709	1.618	1.244	997	0	1.032	0.809	0.920	30	68	0
3D-3D-n6B	1.733	1.610	1.248	994	0	1.704	1.496	1.600	53	91	0
nnn9D-14D-1B	1.655	1.662	1.250	992	0	2.627	2.501	2.564	85	90	0
5D-5D-n2B	1.747	1.606	1.250	992	0	1.806	1.550	1.678	55	67	0
nnn14D-6D-2B	1.790	1.583	1.252	990	0	1.636	1.368	1.502	50	45	0
nnn13D-6D-1B	1.668	1.673	1.260	984	0	3.568	3.410	3.489	115	76	0
nnn14D-14D-1B	1.673	1.672	1.262	983	0	2.637	2.443	2.540	84	96	0
nnn11D-11D-3B	1.701	1.656	1.263	982	0	2.316	2.251	2.283	75	72	0
1D-1D-n2B	1.760	1.623	1.265	980	0	1.371	1.212	1.291	43	56	0
nnn7D-13D-1B	1.681	1.677	1.268	978	0	2.651	2.500	2.576	85	90	0
nnn9D-2D-2B	1.822	1.593	1.270	976	0	1.416	1.053	1.234	41	53	0
nnn5D-5D-3B	1.782	1.619	1.271	976	0	1.925	1.804	1.864	62	44	0
nnn6D-6D-6B	1.744	1.643	1.271	975	0	1.420	1.019	1.220	40	90	0
nnn9D-7D-5B	1.692	1.677	1.272	975	0	1.435	1.272	1.353	45	82	0
nnn13D-13D-5B	1.670	1.696	1.275	973	0	1.325	1.229	1.277	42	97	0
nnn14D-7D-5B	1.706	1.677	1.277	971	0	1.419	1.193	1.306	43	89	0
nnn6D-6D-1B	1.688	1.690	1.278	970	0	3.770	3.709	3.739	123	78	0
nnn2D-7D-6B	1.735	1.661	1.278	970	0	0.939	0.722	0.831	27	78	0
nnn7D-7D-1B	1.691	1.697	1.283	966	0	1.805	1.796	1.800	59	98	0
8D-8D-n1B	1.700	1.692	1.283	966	0	3.076	3.004	3.040	100	95	0
nnn7D-1D-1B	1.733	1.673	1.285	965	0	1.994	1.744	1.869	62	75	0
nnn7D-7D-5B	1.702	1.694	1.286	964	0	1.586	1.473	1.529	50	94	0
4D-4D-n5B	1.719	1.694	1.291	960	0	2.032	2.008	2.020	67	94	0
nnn14D-11D-5B	1.733	1.693	1.296	957	0	1.230	1.029	1.129	37	93	0
nnn9D-9D-2B	1.798	1.654	1.296	956	0	1.234	1.160	1.197	40	63	0
nnn12D-7D-1B	1.742	1.690	1.297	956	0	2.600	2.230	2.415	80	85	0
nnn9D-9D-5B	1.659	1.742	1.297	956	0	1.394	0.000	0.697	23	92	0
1D-1D-n6B	1.750	1.687	1.299	955	0	0.717	0.507	0.612	20	96	0
nnn1D-10D-1B	1.711	1.718	1.302	952	0	2.543	2.396	2.469	81	84	0
nnn2D-2D-2B	1.871	1.619	1.303	952	0	1.438	1.156	1.297	43	48	0
nnn14D-14D-2B	1.790	1.670	1.303	952	0	1.287	1.186	1.237	41	71	0
nnn4D-4D-6B	1.764	1.698	1.310	947	0	0.891	0.800	0.846	28	94	0
n5D-5D-4B	1.838	1.659	1.314	944	0	1.303	0.000	0.651	21	87	0
nnn9D-13D-2B	1.816	1.679	1.318	941	0	1.282	1.187	1.235	41	66	0

nnn3D-12D-5B	1.815	1.685	1.320	939	0	1.390	1.034	1.212	40	75	0
nnn11D-10D-2B	1.811	1.688	1.321	938	0	1.798	1.624	1.711	56	68	0
nnn14D-13D-2B	1.809	1.691	1.322	938	0	1.289	1.191	1.240	41	69	0
nnn9D-11D-5B	1.748	1.733	1.325	936	0	1.355	1.192	1.273	42	82	0
nnn13D-13D-2B	1.817	1.702	1.331	932	0	1.318	1.231	1.274	42	68	0
nnn1D-1D-2B	1.883	1.665	1.334	930	0	1.268	1.073	1.170	39	49	0
nnn12D-12D-1B	1.764	1.744	1.337	928	0	3.102	2.834	2.968	98	97	0
nnn11D-11D-1B	1.755	1.753	1.338	926	0	1.748	1.714	1.731	57	95	0
nnn14D-5D-1B	1.782	1.739	1.340	925	0	2.880	2.501	2.691	89	72	0
nnn9D-3D-5B	1.782	1.740	1.341	925	0	1.528	1.415	1.471	49	97	0
8D-8D-n2B	1.873	1.688	1.343	923	0	2.278	2.025	2.151	71	59	0
nnn6D-5D-2B	1.919	1.660	1.344	923	0	2.143	1.818	1.980	65	43	0
nnn2D-11D-6B	1.816	1.734	1.349	919	0	1.039	0.814	0.926	31	76	0
nnn12D-12D-5B	1.835	1.723	1.350	919	0	1.497	1.167	1.332	44	91	0
8D-8D-n5B	1.779	1.761	1.352	917	0	1.680	1.603	1.642	54	97	0
nnn4D-10D-5B	1.773	1.767	1.353	916	0	1.408	1.352	1.380	46	98	0
nnn11D-5D-6B	1.856	1.733	1.363	909	0	0.978	0.733	0.855	28	67	0
nnn7D-7D-2B	1.872	1.729	1.367	907	0	1.365	1.296	1.330	44	65	0
nnn9D-12D-2B	1.878	1.729	1.369	906	0	1.867	1.691	1.779	59	64	0
nnn1D-7D-1B	1.770	1.797	1.370	905	0	2.673	2.602	2.637	87	89	0
nnn1D-5D-2B	1.947	1.695	1.374	902	0	1.418	1.221	1.319	44	45	0
nnn13D-5D-2B	1.938	1.701	1.375	902	0	2.120	1.654	1.887	62	50	0
nnn2D-2D-1B	1.800	1.802	1.383	897	0	2.522	2.342	2.432	80	84	0
nnn3D-3D-5B	1.834	1.792	1.389	892	0	1.612	1.561	1.587	52	90	0
nnn12D-12D-2B	1.894	1.755	1.390	892	0	1.329	1.217	1.273	42	68	0
nnn12D-5D-1B	1.850	1.783	1.390	892	0	2.862	2.442	2.652	88	70	0
nnn11D-11D-5B	1.822	1.805	1.393	890	0	1.368	1.280	1.324	44	91	0
3D-3D-n5B	1.871	1.779	1.395	889	0	2.059	1.872	1.966	65	87	0
1D-1D-n1B	1.806	1.834	1.404	883	0	2.306	2.241	2.274	75	94	0
nnn11D-7D-2B	1.920	1.780	1.413	877	0	2.058	1.894	1.976	65	66	0
n2D-2D-4B	1.668	1.937	1.414	877	0	1.058	0.000	0.529	17	93	0
nnn5D-5D-2B	2.027	1.733	1.425	870	0	1.629	1.403	1.516	50	42	0
nnn1D-12D-5B	1.916	1.811	1.430	867	0	1.135	0.795	0.965	32	74	0
nnn2D-2D-6B	1.899	1.838	1.439	861	0	0.905	0.692	0.798	26	99	0
nnn11D-11D-2B	1.955	1.817	1.448	856	0	2.665	2.402	2.533	84	68	0
nnn1D-1D-1B	1.873	1.901	1.467	845	0	1.919	1.868	1.893	62	87	0
nnn10D-5D-5B	1.906	1.887	1.470	843	0	1.511	1.407	1.459	48	82	0
nnn2D-13D-5B	1.890	1.900	1.472	842	0	1.468	1.367	1.417	47	91	0
nnn4D-4D-5B	1.903	1.893	1.473	842	0	1.569	1.619	1.594	53	98	0
nnn9D-1D-5B	1.889	1.906	1.475	840	0	1.225	1.151	1.188	39	97	0
2D-2D-n5B	1.956	1.867	1.477	839	0	2.158	2.212	2.185	72	87	0
nnn6D-6D-5B	2.021	1.870	1.502	825	0	2.049	0.000	1.025	34	96	0
nnn5D-5D-1B	1.933	1.926	1.503	825	0	2.735	2.687	2.711	89	72	0
nnn3D-1D-5B	1.943	1.920	1.503	825	0	1.258	1.172	1.215	40	91	0
1D-1D-n5B	1.955	1.998	1.553	799	0	1.121	1.061	1.091	36	95	0
nnn1D-1D-5B	2.054	2.081	1.637	758	0	0.934	0.833	0.884	29	99	0
nnn2D-2D-5B	2.118	2.132	1.689	734	0	1.300	1.246	1.273	42	91	0
nnn5D-5D-5B	2.367	2.186	1.810	685	0	1.593	0.000	0.796	26	88	0
